# In vivo and in vitro expression of steroid-converting enzymes in human breast tumours: associations with interleukin-6

**DOI:** 10.1038/sj.bjc.6690749

**Published:** 1999-10

**Authors:** V Speirs, D S Walton, M-C Hall, S L Atkin

**Affiliations:** Department of Medicine, Medical Research Laboratory, University of Hull, Hull, HU6 7RX, UK

**Keywords:** breast cancer, steroids, interleukin-6

## Abstract

Enzymes modulating local steroid availability play an important role in the progression of human breast cancer. These include isoforms of 17β-hydroxysteroid dehydrogenase (17-HSD), aromatase and steroid sulphatase (STS). The aim of this study was to investigate the expression, by reverse transcription polymerase chain reaction, of 17-HSD types I–IV, aromatase and steroid STS in a series of 51 human breast tumour biopsies and 22 primary cultures of epithelial and stromal cells derived from these tumours, giving a profile of the steroid-regulating network for individual tumours. Correlations between enzyme expression profiles and expression of the interleukin (IL)-6 gene were also sought. All except one tumour expressed at least one isoform of 17-HSD, either alone or in combination with aromatase and STS. Expression of 17-HSD isoforms I–IV were observed in nine tumours. Of the 15 tumours which expressed three isoforms, a combination of 17-HSD II, III and IV was most common (6/15 samples). The majority of tumours (*n* = 17) expressed two isoforms of 17-HSD with combinations of 17-HSD II and IV predominant (7/17 samples). Eight tumours expressed a single isoform and of these, 17-HSD I was in the majority (5/8 samples). In primary epithelial cultures, enzyme expression was ranked: HSD I (86%) > STS (77%) > HSD II (59%) > HSD IV (50%) = aromatase (50%) > HSD III (32%). Incidence of enzyme expression was generally reduced in stromal cultures which were ranked: HSD I (68%) > STS (67%) > aromatase (48%) > HSD II (43%) > HSD IV (28%) > HSD III (19%). Expression of IL-6 was associated with tumours that expressed ≥ 3 steroid-converting enzymes. These tumours were of higher grade and tended to come from patients with family history of breast cancer. In conclusion, we propose that these enzymes work in tandem with cytokines thereby providing sufficient quantities of bioactive oestrogen from less active precursors which stimulates tumour growth. © 1999 Cancer Research Campaign


*British Journal of Cancer* (1999) **81**(4), 690–695
© 1999 Cancer Research Campaign
Article no. bjoc.1999.0749

### In vivo and in vitro expression of steroid-converting enzymes in human breast tumours: associations with interleukin-6

**V Speirs, DS Walton, M-C Hall and SL Atkin**

Department of Medicine, Medical Research Laboratory, University of Hull, Hull HU6 7RX, UK

**Summary** Enzymes modulating local steroid availability play an important role in the progression of human breast cancer. These include isoforms of 17β-hydroxysteroid dehydrogenase (17-HSD), aromatase and steroid sulphatase (STS). The aim of this study was to investigate the expression, by reverse transcription polymerase chain reaction, of 17-HSD types I–IV, aromatase and steroid STS in a series of 51 human breast tumour biopsies and 22 primary cultures of epithelial and stromal cells derived from these tumours, giving a profile of the steroid-regulating network for individual tumours. Correlations between enzyme expression profiles and expression of the interleukin (IL)-6 gene were also sought. All except one tumour expressed at least one isoform of 17-HSD, either alone or in combination with aromatase and STS. Expression of 17-HSD isoforms I–IV were observed in nine tumours. Of the 15 tumours which expressed three isoforms, a combination of 17-HSD II, III and IV was most common (6/15 samples). The majority of tumours ($n$ = 17) expressed two isoforms of 17-HSD with combinations of 17-HSD II and IV predominant (7/17 samples). Eight tumours expressed a single isoform and of these, 17-HSD I was in the majority (5/8 samples). In primary epithelial cultures, enzyme expression was ranked: HSD I (86%) > STS (77%) > HSD II (59%) > HSD IV (50%) = aromatase (50%) > HSD III (32%). Incidence of enzyme expression was generally reduced in stromal cultures which were ranked: HSD I (68%) > STS (67%) > aromatase (48%) > HSD II (43%) > HSD IV (28%) > HSD III (19%). Expression of IL-6 was associated with tumours that expressed ≥ 3 steroid-converting enzymes. These tumours were of higher grade and tended to come from patients with family history of breast cancer. In conclusion, we propose that these enzymes work in tandem with cytokines thereby providing sufficient quantities of bioactive oestrogen from less active precursors which stimulates tumour growth. © 1999 Cancer Research Campaign

**Keywords:** breast cancer; steroids; interleukin-6

Two-thirds of all breast cancers are detected after the menopause, when ovarian oestrogen production has virtually ceased. Despite this, oestrogen levels within breast tumours remain high and it has been demonstrated that these tumours possess enzyme systems required to produce bioactive oestrogens in situ from circulating precursors androstendione or oestrone sulphate (Pasqualini et al, 1996*a*; Peltoketo et al, 1996). In post-menopausal women, these systems are particularly active. The two main routes for oestrogen synthesis within breast tumours from post-menopausal women involve activity of three principal enzyme groups: aromatase, oestrone sulphatase (STS) and 17β-hydroxysteroid dehydrogenase (17-HSD).

The 17-HSD enzyme complex controls the final step in the formation of all androgens and oestrogens, and thus plays a key role in regulating local hormone concentrations (reviewed in Labrie et al, 1997). To date, six distinct isoforms of 17-HSD, designated I–VI, have been identified and cloned. The type I enzyme uses NADPH as a cofactor and catalyses the interconversion of the weak oestrogen, oestrone (E1) to the biologically more potent 17β-oestradiol (E2; Peltoketo et al, 1996; Penning, 1996). Using $NAD^+$ as cofactor, 17-HSD II catalyses the oxidation of testosterone and E2 to form androstendione and E1 respectively (Luu-The et al, 1995; Penning, 1996). This isoform also possesses 20α-hydroxylase activity by converting 20α-dihydroprogesterone to progesterone (Casey et al, 1994). The androgenic 17-HSD III prefers NADPH as cofactor and reduces androstendione to testosterone (as can 17-HSD type I, but to a much lesser extent; Luu-The et al, 1995), and also reduces dehydroepiandrosterone to 5-androstenediol (Andersson, 1995; Luu-The et al, 1995). This isoform is found principally in the testis. Type IV 17-HSD is similar to the type II isoform in that it is $NAD^+$-dependent and is principally involved in the oxidation and therefore inactivation of oestrogens and androgens (Adamski et al, 1992). 17-HSD type V belongs to the aldoketoreductase family (Jornvall et al, 1995) while the sixth isoform is a member of the short-chain alcohol reductase family (Deyashiki et al, 1995). A seventh murine isoform of 17-HSD, present in corpus luteum and which possesses reductive activity has recently been described (Nokelainen et al, 1998).

In addition to 17-HSD, aromatase and STS also contribute to the high local concentrations of E2 observed within breast tumours of post-menopausal women (Reed and Purohit, 1997). Aromatase, a product of the CYP19 gene, catalyses the aromatization of C19 steroids (androstendione, testosterone and 16α-hydroxyandrostendione) to E1 and E2 (Guengerich, 1992; Simpson et al, 1994). It belongs to the cytochrome P450 family of enzymes. STS is responsible for the hydrolysis of oestrone sulphate to oestrone, and

*Received 21 September 1998*
*Revised15 March 1999*
*Accepted 20 April 1999*

*Correspondence to:* V Speirs this pathway is of particular importance in breast tumours as it results in up to ten times more free oestrone than the aromatase pathway (Santer et al, 1984).

As concentrations of oestrogens within breast tumours are significantly higher than plasma levels (Pasqualini et al, 1996) this suggests that in situ production of these hormones is critical. Because of their involvement in the in situ production of E2, the steroid-converting enzymes described above are clearly important in breast cancer progression, but no study to date has addressed the simultaneous expression of all six of these enzymes in breast tumours. Therefore to provide a more precise overview of the expression of gene transcripts for these enzymes in breast cancer, we have determined expression of isoforms I–IV of 17-HSD, aromatase and STS in a series of human breast tumours using reverse transcription polymerase chain reaction (RT-PCR). Further, we have examined expression of these enzymes in primary epithelial and stromal cultures derived from human breast tumours to determine their cellular localization. Finally, as the cytokine IL-6 appears to have a key role in the regulation of aromatase, 17-HSD type STS complexes and I (Adams et al, 1991; Speirs et al, 1993; Purohit et al, 1997) we investigated whether expression of IL-6 was associated with a specific enzyme profile.

**Table 1** Clinicopathological details of the tumours under study

| Parameter | Number |
|---|---|
| Patients | |
| Total number in study | 51 |
| Age (years) | |
| Mean | 62 |
| Range | 35–88 |
| Tumour histology | |
| Ductal | 45 |
| Lobular | 4 |
| Other[a] | 2 |
| Grade | |
| I | 2 |
| II | 23 |
| III | 18 |
| Unknown | 7 |
| Node status | |
| + | 22 |
| – | 23 |
| Unknown | 6 |
| Menopausal status | |
| Pre | 9 |
| Post | 42 |

[a]This group comprised one apocrine and one carcinoma of mixed lobular/ductal pathology

### METHODS

### Tumour samples

Breast tumours were obtained from 51 patients who presented sequentially for surgical removal of a clinically confirmed breast lesion. Tumours were identified and clinically staged using the Nottingham grading system by a consultant pathologist, details of which are presented in Table 1. Surgical samples were immediately snap-frozen and stored at –80°C until analysed. Ethical approval was obtained and all patients gave informed consent.

### RNA extraction and cDNA synthesis

Frozen breast tissue was pulverized using a mortar and pestle and total RNA extracted with Trizol (Life Technologies, Paisley, UK) according to the manufacturer's instructions. One microgram of RNA was used as a template for first strand synthesis as previously described (Green et al, 1997).

### Qualitative PCR amplification

Primers were obtained from Life Technologies and were designed from published gene sequences. Sequences, product sizes and restriction mapping details for all primer pairs are illustrated in Table 2. Details on primers used for amplification of the IL-6 gene have previously been published (Green et al, 1997). To check cDNA integrity, fragments of glyceraldehyde phosphate dehydrogenase (GAPDH), a standard housekeeping gene, were amplified in parallel (data not shown). The PCR reaction contained: 2 units of BIOTAQ, 10 × PCR buffer (containing 1.5 mM magnesium

**Table 2** Primer sequences, expected product sizes and restriction mapping details

| Primer sequence | Product size (bp) | Restriction enzyme | Cleaved products (bp) |
|---|---|---|---|
| 17-HSD I | | | |
| 5′-GTGGACGTGCTGGTGTGTAA-3′ | | | |
| 5′-TGGAAGGTGTGGATGTCCGT-3′ | 389 | Hinf I | 252, 187 |
| 17-HSD II | | | |
| 5′-CTGAGGAATTGCGAAGAACC-3′ | | | |
| 5′-GAAGTCCTTGCTGGCTAACG-3′ | 593 | Pst I | 484, 109 |
| 17-HSD III | | | |
| 5′-ACAATGTCGGAATGCTTCCA-3′ | | | |
| 5′-AGGTTGAAGTGCTGGTCTGC-3′ | 624 | Dde I | 238, 205, 181 |
| 17-HSD IV | | | |
| 5′-TCTATGATGGGTGGAGGATT-3′ | | | |
| 5′-GCGGCGTCCTATTTCCTCAA-3′ | 750 | EcoR I | 595, 155 |
| Aromatase | | | |
| 5′-GCCCCCTCTGAGGTCAAGGAACACA-3′ | | | |
| 5′-CACCCGGTTGTAGTAGTTGCAGGCACT-3′ | 267 | Hinf I | 217, 50 |
| Steroid sulphatase | | | |
| 5′-TCCTACTGTTCTTTCTGTGG-3′ | | | |
| 5′-CTTGCAGTCTCTCAGATTGGT-3′ | 482 | Ava II | 255, 227 |

*© 1999 Cancer Research Campaign*

**Table 3** Co-expression of 17β-hydroxysteroid dehydrogenase (17-HSD) isoforms alone or in combination with aromatase (ARO) and steroid sulphatase (STS) in human breast tumours

| HSD isoform | HSD only | HSD +ARO | HSD +STS | HSD +ARO +STS |
|---|---|---|---|---|
| 4 isoforms (*n* = 9) | 3 | 0 | 3 | 3 |
| 3 isoforms (*n* = 15) | | | | |
| I, II and III | 1 | 1 | 2 | 0 |
| I, II and IV | 3 | 0 | 0 | 0 |
| I, II and IV | 0 | 1 | 0 | 1 |
| II, III and IV | 1 | 1 | 1 | 3 |
| 2 isoforms (*n* = 17) | | | | |
| I and II | 1 | 1 | 1 | 0 |
| I and III | 1 | 0 | 0 | 0 |
| I and IV | 1 | 0 | 0 | 0 |
| II and III | 0 | 0 | 1 | 0 |
| II and IV | 1 | 0 | 5 | 1 |
| III and IV | 1 | 0 | 1 | 2 |
| 1 isoform (*n* = 8) | | | | |
| I | 5 | 0 | 0 | 0 |
| II | 1 | 0 | 0 | 0 |
| III | 0 | 0 | 0 | 0 |
| IV | 1 | 0 | 1 | 0 |
| No isoforms (*n* = 2) | 1 | 1 | 0 | 0 |

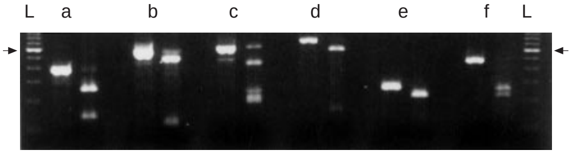

**Figure 1** Representative agarose gel showing original PCR product (left lane) and the fragments resulting from restriction endonuclease digestion (right lane). L = 100 bp marker (Life Technologies), a = 17-HSD I (389), b = 17-HSD-II (593), c = 17-HSD III (624), d = 17-HSD IV (750), e = aromatase (267), f = STS (482). Numbers in parentheses refer to the expected size (bp) of PCR product. Arrowhead = 600 bp

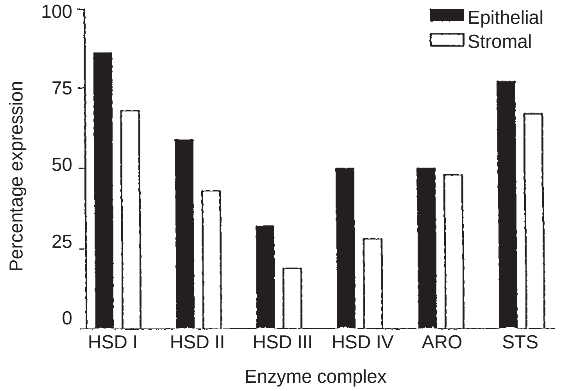

**Figure 2** Relative expression of 17-HSD I-IV, aromatase and STS genes in cultures of epithelial and stromal cells derived from breast tumours. Data is as a percentage (samples which expressed the gene divided by the total number under study)

chloride; both Bioline, London, UK), 0.5 µg of each oligonucleotide primer, 200 µM each of dATP, dCTP, dGTP and dTTP, 1 µl nascent cDNA and sterile distilled water to bring the volume to 50 µl. The following were used as positive controls: for 17-HSD I, II, IV and STS cDNA from the human breast cancer cell line BT-20; for 17-HSD III, human testes cDNA; for aromatase human fibroblast cDNA; for IL-6 human meningioma cDNA (Boyle-Walsh et al, 1996). Negative controls included substitution of RNA or cDNA with distilled water, or substitution of cDNA with an irrelevant cDNA (synthesized from human tibialis anterior muscle). All transcripts were analysed in parallel on at least two separate occasions in a thermal cycler (Hybaid OmniGene, Teddington, UK) with the following cycle: a denaturation step at 94°C for 2 min, followed by 35 cycles of 94°C for 30 s, 57.5°C for 30 s, 72°C for 30 s and a final primer extension step of 72°C for 5 min. PCR products were analysed by electrophoresis through a 1.2% agarose gel and visualized by ethidium bromide staining under UV illumination.

### Restriction enzyme digests

Restriction digests were performed on representative PCR products to confirm the identity of the PCR products. Five microlitres of amplified product was digested with the appropriate restriction enzyme (see Table 2) for 2 h at 37°C. Digested products were then electrophoresed through a 2% agarose gel and visualized as above.

### Primary cultures

Epithelial or stromal-enriched cultures were prepared from 22 breast tumours. Tumours were digested overnight in 0.1% collagenase III (Life Technologies; Speirs et al, 1996*a*). The tissue digest was separated into epithelial or stromal fractions by differential centrifugation followed by culture in selective medium as previously described (Speirs et al, 1996*b*, 1998*a*). Cell were lysed in situ by the addition of Trizol directly to the culture dishes and the resulting RNA reverse transcribed into cDNA, which was amplified by PCR as outlined above.

### Statistical analysis

Differences were calculated using Fisher's Exact test.

### RESULTS

### Expression of genes for steroid converting enzyme in breast tumours

Combinations of steroid-converting enzymes expressed by breast tumours were analysed and compared. The results are presented in Table 3. All except a single tumour expressed at least one isoform of 17-HSD either alone or in combination with aromatase or STS. Nine tumours expressed all four isoforms of 17-HSD, with 3/9 additionally expressing STS and a further 3/9 co-expressing STS and aromatase. Fifteen tumours expressed three 17-HSD isoforms. Of those which exclusively expressed 17-HSD, a combination of 17-HSD II, III and IV was most common, observed in 6/15 samples. Where 17-HSD was expressed in combination with aromatase and STS, combinations of 17-HSD II, III and IV were most common (6/15 samples). The majority of tumours expressed two isoforms of 17-HSD with combinations of 17-HSD II and IV predominant. Eight samples expressed a single isoform of which 17-HSD I, expressed by 5/8 samples was most common. Only one tumour failed to express either one of the 17-HSD isoforms, aromatase or STS and one sample expressed aromatase only. Representative PCR products and restriction-mapped products to confirm identity are illustrated in Figure 1.

© 1999 Cancer Research Campaign

**Table 4** Associations in expression of 17β-hydroxysteroid dehydrogenase (17-HSD) isoforms, aromatase (ARO) and steroid sulphatase (STS) with IL-6 in human breast tumours

| HSD isoform | HSD only | | HSD +ARO | | HSD +STS Interleukin-6 | | HSD +ARO+STS | |
|---|---|---|---|---|---|---|---|---|
| | + | – | + | – | + | – | + | – |
| 4 isoforms (*n* = 9) | 2[a] | 1 | 0 | 0 | 2 | 1 | 3 | 0 |
| 3 isoforms (*n* = 15) | 3 | 2 | 2 | 0 | 3 | 0 | 4 | 1 |
| 2 isoforms (*n* = 15) | 2 | 3 | 0 | 1 | 3 | 5 | 1 | 0 |
| 1 isoforms (*n* = 8) | 3 | 4 | 0 | 0 | 1 | 0 | 0 | 0 |
| No isoforms (*n* = 2) | 1 | 0 | 0 | 1 | 0 | 0 | 0 | 0 |

[a]Each entry represents the number of samples expressing a particular gene combination.

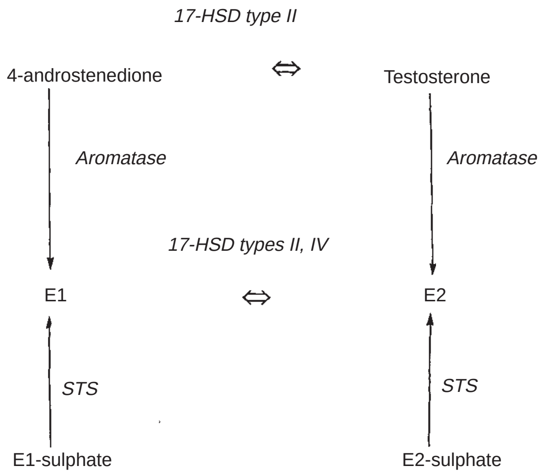

**Figure 3** Schematic representation of the relationship between 17-HSD types II and IV, aromatase and STS. Enzyme pathways are represented in italics. Arrows refer to preferred direction of enzyme. E1 = 17β-oestradiol, E2 = oestrone, STS = steroid sulphatase

### Expression of genes for steroid-converting enzyme in breast primary cultures

To determine if expression of specific genes was associated with a specific cell type, paired epithelial or stromal primary cultures were established from 22 breast tumours. The phenotypic identity of these cultures (all used at passage 1) has previously been characterized by immunostaining, flow cytometry and gene expression studies (Speirs et al, 1996*b*, 1998). Data are illustrated in Figure 2. In epithelial cultures, expression was ranked: HSD I (86%) > STS (77%) > HSD II (59%) > HSD IV (50%) = aromatase (50%) > HSD III (32%). With the exception of aromatase, expression in stromal cultures was generally reduced and was ranked: HSD I (68%) > STS (67%) > aromatase (48%) > HSD II (43%) > HSD IV (28%) > HSD III (19%).

### Correlation between 17-HSD isoforms, aromatase and STS and IL-6 gene expression

Samples, which expressed combinations of 17-HSD isoforms, aromatase and STS with IL-6, were analysed and compared. As illustrated in Table 4, samples which expressed three or more steroid-converting enzymes also tended to express IL-6. These samples tended to be of a higher grade and, additionally had a family history of breast cancer. No further relationships were observed between enzyme profiles and other clinical features including tumour type, stage or grade node or steroid receptor status (data not shown).

### DISCUSSION

Clinical, epidemiological and laboratory data indicate that oestrogens are the most important mitogenic stimulants in breast cancer and local production of oestrogen is of significance in the progression of this disease. Three main steroid-converting enzyme complexes, namely, aromatase, 17-HSD and STS, govern in situ production of oestrogen. In this study we have investigated the presence and distribution of these enzyme complexes in human breast cancer and their association with the cytokine IL-6, which is emerging as having a key role in regulating oestrogen synthesis in breast cancer.

Patterns of enzyme expression were highly variable between individual tumours. However, the majority of samples expressed either two (*n* = 17) or three (*n* = 15), isoforms of 17-HSD, either alone or in combination with aromatase or STS. Since 17-HSD type I is the principal enzyme in breast tissue, catalysing the reduction of the weaker oestrogen, oestrone (E1) into E2 (Peltoketo et al, 1996), one might predict that this isoform would be present in most of these samples. However, this was not the case. In tumours expressing three isoforms, combinations of 17-HSD types II, III and IV were most common, found in 40% of samples. Similarly, in samples expressing two isoforms, combinations of 17-HSD types II and IV predominated (41% of samples). To explain these findings, the specific functions of the steroid-converting enzymes under study should be considered. 17-HSD type II oxidizes testosterone and E2 into androstendione and E1 (Luu-The et al, 1995; Penning, 1996). The function of 17-HSD type IV is similar in that it oxidizes oestrogens and androgens (Adamski et al, 1992). Much of the E1 formed from androstendione is converted by STS to oestrone sulphate, which acts as a reservoir for the formation of E1 via STS (Reed et al, 1994). One could speculate that to produce the biologically more potent E2 associated with breast tumours, the aromatase and STS complexes then take over by converting testosterone or oestrone sulphates into E2. In those tumours lacking 17-HSD type I, this would circumvent the need for this isoform. A schematic representation showing the proposed relationship between 17-HSD types II and IV, aromatase and STS is illustrated in Figure 3.

An unexpected observation was the presence of transcripts for 17-HSD type III in breast tissue. This isoform is found principally in tests where it reduces androstendione to testosterone (Andersson, 1995). Unlike the isoforms for type I, II and IV, which have been detected in a variety of tissue types (Penning, 1996), other groups have failed to detect the 17-HSD type III gene in either ovary (Zhang et al, 1996), or prostate (Elo et al, 1996; Castagnetta et al, 1997). However, it has recently been detected in human adipose tissue (Corbould et al, 1998). Given that human breast tissue contains adipocytes, one might predict that its expression may be associated with these cells. However, we also observed the gene in breast primary cultures. These cultures have been fully characterized using a number of cellular and genetic markers and under the culture conditions described are unlikely to contain adipocytes (Speirs et al, 1996*b*, 1998*a*). Furthermore, the observation of 17-HSD type III expression in human pituitary adenomas (Green et al, 1999) and in a human colonic carcinoma cell line (English et al, 1997) lends further support for an active role of 17-HSD type III in steroid metabolism.

Like all studies which evaluate gene expression, PCR gives no indication of whether the gene transcripts go on to be translated and transcribed into bioactive enzyme in breast tumours. Nevertheless there is good evidence that these enzyme complexes are active in tumours and isolated cell types. We have recently reported a correlation between gene expression and enzyme activity for 17-HSD type I in epithelial and stromal cells cultured derived from breast tumours (Speirs et al, 1998*b*) and pituitary adenomas (Green et al, 1999). Similar correlations have been observed for STS and aromatase (Lu et al, 1996; Pasqualini et al, 1996*b*; Sasano et al, 1996). In a previous study, we have confirmed by ELISA that the IL-6 gene is translated and secreted as a bioactive peptide in breast primary cultures (Green et al, 1997).

In general, our in vitro data showed that genes for steroid-converting enzymes were more likely to be expressed by epithelial cells compared with stromal cells. Of the six genes analysed, 17-HSD I and STS were observed in a greater number of samples (in epithelial cultures, 86% and 77% respectively). Although it is possible that removing a cell from its internal microenvironment and placing it under in vitro conditions may alter its phenotypic characteristics, our data correlates with previous studies looking at enzyme activity. These studies showed that 17-HSD I (Speirs et al, 1998) and STS (James et al, 1987) had highest levels of activity in breast tumours. Further, with aromatase we observed the gene in approximately 50% of samples, complementing an earlier study on aromatase activity in breast tumours (Tilson-Mallet et al, 1983). Immunostaining data reveals that the aromatase enzyme is located in both epithelial and stromal cells (Santen et al, 1994; Lu et al, 1996; Sasano et al, 1996).

Cytokines have emerged as key regulators of E2 synthesis in the breast, in particular IL-6. As IL-6 can stimulate the activity of 17-HSD, aromatase and STS (Adams et al, 1991; Speirs et al, 1993; Purohit et al, 1997), we investigated whether there was a relationship between expression of IL-6 with 17-HSD isoforms, aromatase and STS. In general, samples which expressed at least three isoforms of 17-HSD in combination with IL-6 tended to be of higher grade (either grades II or III). Those which expressed all six enzymes in combination with IL-6 were of grade III. Further analysis revealed that tumours which expressed IL-6 and enzyme combinations which included HSD I, aromatase and STS came from patients with grade III carcinomas and from those with a family history of breast cancer (first-degree relative). These enzymes are regarded as being most critical in regulating oestrogen concentrations in breast cancer, and furthermore their activities are significantly up-regulated by IL-6 in vitro (Purohit et al, 1997). Our observation that these factors are associated with more aggressive tumours could indicate that co-expression of IL-6 with HSD type I, aromatase and STS may be useful as independent prognostic factors in breast cancer.

It is worth noting that paradoxically, whilst IL-6 stimulates oestrogen production via these enzyme complexes, oestrogen itself can down-regulate IL-6 at the transcriptional level (Ray et al, 1994; Kurebayashi et al, 1997). These inhibitory effects appear to be indirect, interfering with the function of the transcription factor NF-κB (Kurebayashi et al, 1997). Furthermore, as aberrant expression of NF-κB has been observed in many breast tumours (Sovak et al, 1997), the inhibitory effects of oestrogens on IL-6 transcription may be lost.

In conclusion, we have shown that breast tumours show considerable heterogeneity in expression of steroid-converting enzymes. We propose that these enzymes work in tandem, probably in association with cytokines, thereby providing sufficient quantities of bioactive oestrogen from less active precursors which stimulates tumour growth. Detection of these enzymes in cultures enriched for stromal as well as epithelial cells could indicate modulation of enzyme activity by the stroma. This may represent a novel paracrine mechanism which increases availability of E2 to breast tumours.

### ACKNOWLEDGEMENTS

We thank Messrs PJ Carleton and JN Fox for kindly providing us with breast tumour samples and are grateful to Dr E Gardiner, Hull University Statistical Support Unit for advice on statistics. This work was supported by Yorkshire Cancer Research and Royal Hull Hospitals NHS Trust.

### REFERENCES

Adams EF, Rafferty B and White MC (1991) Interleukin 6 is secreted by breast fibroblasts and stimulates 17β-oestradiol oxidoreductase activity in MCF-7 cells: possible paracrine regulation of 17β-oestradiol levels. *Int J Cancer* **49**: 118–121

Adamski J, Normand T, Leenders F, Monte D, Begue A, Stehelin D, Jungblut PW and de Launoit Y (1992) Molecular cloning of a novel widely expressed human 80 kDa 17β-hydroxysteroid dehydrogenase IV. *Biochem J* **311**: 437–443

Andersson S (1995) 17β-hydroxysteroid dehydrogenase: isozymes and mutations. *J Endocrinol* **146**: 197–200

Boyle-Walsh E, Birch MA, Gallagher J, Speirs V, White MC, Shenkin A and Fraser WD (1996) RT-PCR detection of cytokine transcripts in a series of cultured human meningiomas. *J Pathol* **178**: 442–446

Casey ML, Macdonald PC and Andersson S (1994) 17β-hydroxysteroid dehydrogenase type 2: chromosomal assignment and progestin regulation of gene expression in human endometrium. *J Clin Invest* **94**: 2135–2141

Castagnetta LAM, Carruba G, Traina A, Granata OM, Markus M, Pavone-Macaluso M, Blomquist CH and Adamski J (1997) Expression of different 17β-hydroxysteroid dehydrogenase types and their activities in human prostate cancer cells. *Endocrinology* **138**: 4876–4882

Corbould AM, Judd SJ and Rodgers RJ (1998) Expression of types 1, 2 and 3 17β-hydroxysteroid dehydrogenase in subcutaneous abdominal and intra-abdominal adipose tissue of women. *J Clin Endo Metab* **83**: 187–194

Deyashiki Y, Ohshima K, Nakanishi M, Sato K, Matsuura K and Hara A (1995) Molecular cloning and characterisation of mouse estradiol 17β-dehydrogenase (A-specific), a member of the aldoketoreductase family. *J Biol Chem* **270**: 10461–10467

Elo JP, Akinola LA, Poutanen M, Vihko P, Kyllonen AP, Lukkarinen O and Vihko R (1996) Characterisation of 17β-hydroxysteroid dehydrogenase isoenzyme expression in benign and malignant human prostate. *Int J Cancer* **66**: 37–41

English M, Robinson E, Hughes SV, Morton D, Langman MJ, Stewart PM and Hewison M (1997) Demonstration in vitro and in vivo of oestrogen metabolism in colonic cancer tissue. *J Endocrinol Suppl* **152**: P95.

Green AR, Green VL, White MC and Speirs V (1997) Expression of cytokine messenger RNA in normal and neoplastic human breast tissue: identification of interleukin-8 as a potential regulatory factor in breast tumours. *Int J Cancer* **72**: 937–941

Green VL, Speirs V, Jeffreys RV, Foy P, Landolt AM and Atkin SL (1999) 17β-hydroxysteroid dehydrogenase type 1, 2, 3 and 4 expression and enzyme activity in human anterior pituitary adenomas. *J Clin Endo Metab* **84**: 1340–1345

Guengerich FP (1992) Characterisation of human cytochrome P450 enzymes. *FASEB J* **6**: 745–748

James VHT, McNeill JM, Lai LC, Newton CJ, Ghilchik MW and Reed MJ (1987) Aromatase activity in normal breast and breast tumour tissue: in vivo and in vitro studies. *Steroids* **50**: 269–279

 © 1999 Cancer Research Campaign

Jornvall H, Person B, Krook M, Atrain S, Gonzalez-Duatre R, Jeffrey J and Ghosh D (1995) Short-chain dehydrogenases/reductases (SDR). *Biochem* **34**: 6003–6013

Kurebayashi S, Miyashita Y, Hirose T, Kasayama S, Akira S and Kishimoto T (1997) Characterisation of mechanisms of interleukin-6 repression by estrogen receptor. *J Steroid Biochem Mol Biol* **60**: 11–17

Labrie F, Luu-The V, Lin S-X, Labrie C, Simard J, Breton R and Belanger A (1997) The key role of 17β-hydroxysteroid dehydrogenases in sex steroid biology. *Steroids* **62**: 148–158

Lu Q, Nakmura J, Savinov A, Yue W, Weisz J, Dabbs DJ, Wolz G and Brodie A (1996) Expression of aromatase protein and messenger ribonucleic acid in tumour epithelial cells and evidence of functional significance of locally produced estrogen in human breast cancers. *Endocrinology* **137**: 3061–3068

Luu-The V, Zhang Y, Poirier D and Labrie F (1995) Characteristics of human types 1, 2 and 3 17β-hydroxysteroid dehydrogenase activities: oxidation/reduction and inhibition. *J Steroid Biochem Molec Biol* **55**: 581–587

Nokelainen PA, Peltoketo H, Vihko RK and Vihko PT (1998) Expression cloning of a novel mouse 17β-hydroxysteroid dehydrogenase/17-ketosteroid reductase, an enzyme of estradiol biosynthesis in the corpus luteum. *Proc Am Endo Soc* P1–279

Pasqualini JR, Chetrite G, Blacker C, Feinstein MC, Delonde L, Talbi M and Maloche C (1996*a*) Concentrations of estrone, estradiol and estrone sulfate and evaluation of sulfatase and aromatase activities in pre- and post-menopausal breast cancer patients. *J Clin Endocrinol Metab* **81**: 1460–1464

Pasqualini JR, Chetrite G and Le Nestour E (1996*b*) Control and expression of oestrone sulphatase activities in human breast cancer. *J Endocrinol* **150**: S99–S105

Peltoketo H, Isomaa V and Vihko R (1996) Expression and regulation of 17β-hydroxysteroid dehydrogenase type I. *J Endocrinol* **150**: S21–S30

Penning TM (1996) Molecular endocrinology of hydroxysteroid dehydrogenases. *Endocrine Rev* **18**: 281–305

Purohit A, Duncan LJ, Wang DY, Coldham NG, Ghilchik MW and Reed MJ (1997) Paracrine control of oestrogen production in breast cancer. *Endocrine Rel Cancer* **4**: 323–330

Ray A, Prefontaine KE and Ray P (1994) Down-modulation of interleukin-6 gene by 17β-estradiol in the absence of high affinity DNA binding by the estrogen receptor. *J Biol Chem* **269**: 12940–12946

Reed MJ and Purohit A (1997) Breast cancer and the role of cytokines in regulating estrogen synthesis: An emerging hypothesis. *Endo Rev* **18**: 701–705

Reed MJ, Purohit A, Howarth NM and Potter BVL (1994) Steroid sulphatase inhibitors: a new endocrine therapy. *Drugs Future* **19**: 673–680

Santen RJ, Martel J, Hoahland F, Naftolin F, Roa L, Harada N, Hafer L, Zaino R and Santer SJ (1994) Stromal spindle cells contain aromatase in human breast tumours. *J Clin Endo Metab* **79**: 627–632

Santer SJ, Feil PD and Santen RJ (1984) In situ estrogen production via the estrone sulphatase pathway in breast tumours: relative importance versus the aromatase pathway. *J Clin Endo Metab* **59**: 29–33

Sasano H, Frost AR, Saitoh R, Harada N, Poutanen M, Vihko R, Bulun SE, Silverberg SG and Nagura H (1996) Aromatase and 17β-hydroxysteroid dehydrogenase type I in human breast carcinoma. *J Clin Endo Metab* **81**: 4042–4045

Simpson ER, Mahendroo MS, Means GD, Kilgore MW, Hinshelwood MM, Graham-Lorence S, Amarneh B, Ito Y, Fisher CR, Michael MD, Mendelson CR and Bulun SE (1994) Aromatase cytochrome P450, the enzyme responsible for estrogen biosynthesis. *Endo Rev* **15**: 342–355

Sovak MA, Bellas RE, Kim DW, Zanieski GJ, Rogers AE, Traish AM and Sonenshein GE (1997) Aberrant nuclear factor-kappaB/Rel expression and the pathogenesis of breast cancer. *J Clin Invest* **100**: 2952–2960

Speirs V, Adams EF, Rafferty B and White MC (1993) Interactive effects of interleukin-6, 17β-estradiol and progesterone on growth and 17β-hydroxysteroid dehydrogenase activity of human breast carcinoma cells. *J Steroid Biochem Mol Biol* **46**: 11–15

Speirs V, Green AR and White MC (1996*a*) Collagenase III: a superior enzyme for complete disaggregation and improved viability of normal and malignant human breast tissue. *In Vitro Cell Dev Biol* **32**: 72–74

Speirs V, Green AR and White MC (1996*b*) A comparative study of cytokine gene transcripts in normal and malignant human breast tissue and primary cell cultures derived from the same tissue samples. *Int J Cancer* **66**: 551–556

Speirs V, Green AR, Walton DS, Kerin MJ, Fox JN, Carleton PJ, Desai SB and Atkin SL (1998*a*) Short-term primary culture of epithelial cells derived from breast tumours. *Br J Cancer* **78**: 1421–1429

Speirs V, Green AR and Atkin SL (1998*b*) Expression and activity of 17β-hydroxysteroid dehydrogenase type I in primary cultures of epithelial and stromal cells derived from normal and tumourous human breast tissue: The role of IL-8. *J Steroid Biochem Mol Biol* **67**: 267–274

Tilson-Mallet N, Santer SJ, Feil PD and Santen RJ (1983) Biological significance of aromatase activity in human breast tumours. *J Clin Endo Metab* **57**: 1125–1128

Zhang Y, Word RA, Fesmire S, Carr BR and Rainey WE (1996) Human ovarian expression of 17β-hydroxysteroid dehydrogenase types 1, 2 and 3. *J Clin Endo Metab* **81**: 3594–3598

*© 1999 Cancer Research Campaign*
